# How epidemics end

**DOI:** 10.1111/1600-0498.12370

**Published:** 2021-02-22

**Authors:** Erica Charters, Kristin Heitman

**Affiliations:** ^1^ University of Oxford Oxford UK; ^2^ Independent Scholar Bethesda Maryland USA

**Keywords:** cholera, COVID‐19, disease modelling, endemic disease, epidemics, plague

## Abstract

As COVID‐19 drags on and new vaccines promise widespread immunity, the world's attention has turned to predicting how the present pandemic will end. How do societies know when an epidemic is over and normal life can resume? What criteria and markers indicate such an end? Who has the insight, authority, and credibility to decipher these signs? Detailed research on past epidemics has demonstrated that they do not end suddenly; indeed, only rarely do the diseases in question actually end. This article examines the ways in which scholars have identified and described the end stages of previous epidemics, pointing out that significantly less attention has been paid to these periods than to origins and climaxes. Analysis of the ends of epidemics illustrates that epidemics are as much social, political, and economic events as they are biological; the “end,” therefore, is as much a process of social and political negotiation as it is biomedical. Equally important, epidemics end at different times for different groups, both within one society and across regions. Multidisciplinary research into how epidemics end reveals how the end of an epidemic shifts according to perspective, whether temporal, geographic, or methodological. A multidisciplinary analysis of how epidemics end suggests that epidemics should therefore be framed not as linear narratives—from outbreak to intervention to termination—but within cycles of disease and with a multiplicity of endings.

As COVID‐19 drags on and candidate vaccines seem to promise widespread immunity, the world's attention has turned to predicting how the present pandemic will end. Many such predictions invoke historical antecedents—typically the plague, smallpox, the 1918 influenza, and SARS.^1^ Others make use of mathematical models to trace the downward epidemic (or “epi”) curve and thereby forecast end dates.^2^ Yet the end of an epidemic includes not only the decline of disease incidence and associated deaths, but also the lifting of public health regulations and the associated political, social, and economic restrictions. The formal end of an epidemic thus signals a return to normal life. For example, the multinational pharmaceutical company AstraZeneca has committed to providing globally its Oxford‐AstraZeneca COVID‐19 vaccine on a not‐for‐profit basis for the duration of the COVID‐19 pandemic.^3^ The end of the epidemic, therefore, will mean its return to normal business practices, as it will for other businesses.

Yet how do societies know when an epidemic has ended and normal life can resume? What criteria and markers indicate an epidemic's end? Who has the insight, authority, and credibility to decipher these signs? Although researchers have paid a great deal of attention to the origins of epidemics and to the climactic high points of outbreaks, they have paid little attention to how epidemics actually end. The detailed research that has been conducted demonstrates that epidemics do not end suddenly. Indeed, few epidemic diseases are eradicated. Far more often, an epidemic is declared to have ended once the disease falls to endemic levels, when it becomes an accepted, manageable part of normal life in a given society. In other words, contrary to popular assumption and idealistic hope, epidemics do not generally end through the abrupt eradication of a disease or the quick administration of a vaccine. While some, such as recent outbreaks of cholera, have been ended through medical, social, and political interventions, others, including influenza and HIV/AIDS, have never ended. Instead, they persist as cyclical epidemics or endemic diseases.

Standard histories that do describe an ending tend to attribute the end to a single, straightforward, often rapid change: a shift in the dominant rodent species to explain the disappearance of plague, the removal of the handle of the Broad Street pump to stop a cholera outbreak in London, or the clever distribution of an effective vaccine to eliminate smallpox. This narrative habit exemplifies what historians and sociologists of science have observed about the crystallization of a single master‐narrative to make retroactive sense of complex processes.^4^ Such accounts reflect a deeply felt need for intelligible explanations that consist of a narrative arc with a clear origin, progression, and end.

This article examines the ways in which scholars have identified and described the end stages of past epidemics, pointing out that significantly less attention has been paid to the “ending” of epidemics than to their origins and climax. Research demonstrates that the course of any epidemic is best gauged through several overlapping measures and conditions. Equally important, epidemics end at different times for different groups, both within one society and across regions. These groups not only experience the social, economic, and political effects of a disease differently, but are subject to significantly different forces and conditions, particularly over time. Moreover, analysis of the ends of epidemics illustrates how epidemics end through a process of negotiation between biological factors, on the one hand, and political and social interests, on the other. Whereas the outbreak of an epidemic centres attention on the disease itself, attention to its ending reveals the nature of epidemics as social and political events, and not simply a biological phenomenon. More generally, it is only by understanding how an epidemic has ended that the origin and course of the outbreak can be fully comprehended.

This article provides a framework to encourage multidisciplinary research into how epidemics end.^5^ This new framework reveals that the end of an epidemic shifts according to perspective. Not only does a given epidemic “end” at different times in different locations, and for different groups in the same location, but also for different academic disciplines: epidemiologists, anthropologists, policymakers, and historians follow different parameters to gauge the decline and end of epidemics. A multidisciplinary analysis of how epidemics end therefore suggests that epidemics should be framed not within linear narratives—from outbreak to intervention to termination—but within cycles of disease and with a multiplicity of endings.

## 1 NARRATIVES AND ENDS

As historians of disease have long pointed out, accounts of an epidemic are typically presented in a standard narrative formulation, which often shapes the telling and interpretation of each new outbreak.^6^ In a seminal series of articles, historian of medicine Charles Rosenberg framed epidemics as social phenomena that unfold in a narrative and dramaturgic form: “Epidemics start at a moment in time, proceed on a stage limited in space and duration, following a plot line of increasing and revelatory tension, move to a crisis of individual and collective character, then drift toward closure.”^7^ That sense of drift seems largely due to the identification of an epidemic's end as the point when the urgency of the disease outbreak has sufficiently diminished so that public attention is redirected to the moral and social crises that the disease has engendered or exposed.^8^ As historians of medicine Jeremy Greene and Dora Vargha remark,

[t]he last part, the end of an epidemic, is perhaps always ever an asymptote, never disappearing but rather fading to the point where its signal is lost in the noise of a new normal, and even allowed, in some imaginable future, to be forgotten.^9^

Thucydides's foundational account of the plague of Athens—the plague of 430 BCE—epitomizes the narrative inattention to an epidemic's end. Despite extensive debates about what disease Thucydides describes and whether its mortality rates and social consequences can be accurately determined, other epidemic narratives that invoke reports of previous epidemics often depend on Thucydides's account in making comparisons and assessments of their own.^10^ As historian Paul Slack has explained,

one can never be entirely sure about the extent to which chroniclers of epidemics concentrated on social dislocation, the failure of doctors, flights to and from religion, rumours of poisoned wells, and similar phenomena simply because Thucydides and later writers down to Defoe *taught them to look for them*.^11^

After graphically describing the disease and its devastating political and social effects, Thucydides simply stops mentioning it. To be clear: he does not mention a decline in cases, nor even the process of returning to normality. Instead, the disease simply disappears from his narrative. We know that the epidemic is over only because it is no longer part of the plot.^12^ Instead, Thucydides returns to describing the progress of the war that plague had interrupted.

This narrative pattern is repeated in other accounts and scholarly studies. Compared with the mass of research done on the origin or causes of epidemics and the details of their course, far less attention is paid to when, how, and why they ended. Rosenberg's own history of cholera in 19th‐century America does not discuss an ending in any detail. Likewise, Alfred Crosby's influential history of the 1918 influenza pandemic has a final chapter tantalizingly titled “Where Did the 1918 Flu Go?”—yet he spends far more time outlining theories of the epidemic's origins than in delineating its end.^13^ Even in comprehensive histories that incorporate multidisciplinary understandings of disease, such as Frandsen's outstanding 500‐page *The Last Plague in the Baltic Region*, brief accounts of an epidemic's end are overshadowed by detailed analysis of its origins and unfolding.^14^

When discussion of an end does not appear as a brief afterword, it forms an equally cursory opening to a chapter titled “Lessons Learned,” in which attention already pivots away from the end itself.^15^ This pattern also holds in fields other than history. As a recent epidemiological modelling paper points out, “While many works have focused on the growth, peak and controlled phases of epidemics,” much less studied are the “tail end dynamics” of an epidemic.^16^ As a result, “there is still much we do not know about the dynamics of an outbreak as it approaches its end.”^17^ Statistical charts of cases—the epidemic (or “epi”) curve ubiquitous in visualizations of an epidemic's “lifecycle”—provide an underlying temporal framework that mirrors Rosenberg's linear narrative of origin, climax, and drift to closure. Such studies have likewise overwhelmingly focused on modelling transmission dynamics and the growth and peak of epidemic curves, neglecting what is commonly called the “control” phase of decline and termination.^18^

In many ways, this lack of attention to the end stages simply reflects the very definition of the term “epidemic.” An epidemic is clinically defined as an “increase, often sudden, in the number of cases of a disease above what is normally expected,” which in turn warrants official attention, if not designation as a “public health emergency of international concern.”^19^ The stages of the epidemic's lifecycle that present themselves for study and reporting are therefore the outbreak, growth, and climax. By contrast, when the crisis recedes and the disease is no longer a cause for alarm, a society's attention can be directed elsewhere.

## 2 LOCATING AND DEFINING ENDS

Accounts of an epidemic's end must therefore be teased out from a paucity of primary sources and academic research. Some describe the “mop‐up stage” at the end of an epidemic as a crucial but hidden end measure, akin to the period when firefighters have extinguished the main blaze but still need to identify and snuff out smouldering ashes.^20^ Evidence for the end of an epidemic may be found in its sequelae (the medical term for conditions and diseases that occur as a result of another disease), especially since the end of an epidemic can also be a turn to addressing a new set of health problems resulting from the epidemic disease.^21^ Even when official declarations of the end to an epidemic are recorded, they conceal the negotiations between measurements of disease rates, on the one hand, and measures of mounting social and economic distress (and political pressures to return to normal life), on the other.^22^ The end of an epidemic is therefore more accurately categorized as a process, rather than a single decisive event.

Recent epidemics of the Ebola virus disease demonstrate the drawn‐out and contentious process of an epidemic's end. The World Health Organization (WHO) has been criticized both for being slow to declare outbreaks of Ebola a public health emergency of international concern (PHEIC), and for terminating such declarations too soon, thereby releasing agencies and donors “to focus their energies elsewhere.”^23^ But as the WHO recognized, and many others have detailed, the termination intended in these declarations was by no means the end of the profound social, political, and economic crises that the epidemics had engendered.^24^ The WHO insists that its authority—particularly in making declarations—is specific to PHEIC events. In other words, its declarations are not about disease or even epidemics per se, but about what constitutes a public health emergency that should concern other nations through the risk of a global outbreak or the need for international assistance.^25^ As a consequence, the WHO did not declare a PHEIC in the midst of the 2019 Ebola outbreak in the Democratic Republic of Congo, not because there were no cases of Ebola, but because it foresaw no international implications that would require the declaration of “a PHEIC at that stage.”^26^

Formal declarations of an end can prove false. Twice in 2015, cases of Ebola were observed in Liberia after declarations of the end of the outbreak. In other words, the WHO made a total of three declarations of Ebola's end, each just months after previously claiming the outbreak was over. Not surprisingly, the WHO's language shifted in each end‐declaration. After the initial declaration that Liberia was “Ebola free” in May, the second declaration in September more soberly reported an end to “Ebola transmission in Liberia.”^27^ Ebola was again identified 2 months later. The WHO cautiously announced in January 2016 that the “latest Ebola outbreak” in Liberia was over, adding that “more flare‐ups are expected.”^28^ As one news source reported, at this third end‐declaration there were “no signs of celebration such as the ‘Ebola free’ T‐shirts that people wore after previous WHO announcements.”^29^ The WHO declared the official end of West Africa's PHEIC for Ebola on March 29, 2016. As its declaration clarified, this was not the end of the disease. Instead, the WHO explained that “Ebola transmission in West Africa no longer constitutes an extraordinary event, that the risk of international spread is now low, and that countries currently have the capacity to respond rapidly to new virus emergences.”^30^

Although the WHO marks a particularly modern and international form of public health authority, its end‐declarations draw on a long history of distinguishing between the end of a crisis and the absence of disease. In August 2010, for example, the WHO declared that the 2009 H1N1 (swine flu) pandemic was in its “post‐pandemic period”; explaining that “cases and outbreaks due to the H1N1 (2009) virus are expected to occur” throughout the post‐pandemic period according to “seasonal patterns of influenza.”^31^ Likewise, medieval Arab medical texts distinguish between widespread (epidemic) outbreaks and individual cases of disease.^32^ In April 1712, Frederick IV of Denmark declared the end of a plague epidemic through a national day of prayer and thanksgiving and the reopening of city gates, even though cases of plague were still observed.^33^ In England, the end of its many 19th‐century cholera epidemics was most often framed through the declaration of a “general thanksgiving to Almighty God upon the great decrease of cholera,” others even using the terminology of “abatement.”^34^ Such language accurately captured the reality that these days of thanksgiving were about the decline, and not end, of cholera; in the week when Queen Victoria declared a national day of “General Thanksgiving,” 11 deaths from cholera were recorded in London alone. *The Times* clarified that this number marked a sharp decline from previous months of “cholera‐deaths, so lately counted by hundreds and thousands.”^35^

There are longstanding formal reasons for this approach. John Graunt (1620–1674), widely viewed as the founder of epidemiology, established the core metrics and overall pattern of an epidemic by using 16th‐ and 17th‐century London mortality counts to identify and compare the dates of outbreak, peak, and ending of several episodes of plague. He first calculated periodic mortalities ascribed to plague, then showed how to correct them, and finally demonstrated the concept of “excess deaths.”^36^ He held explicitly that a plague epidemic ended not when the plague was gone, but when the count of plague deaths returned to the non‐zero “normal” rate established by reporting in more ordinary times.^37^ In this respect, Graunt originated a new narrative form for epidemics, similar to Rosenberg's dramaturgic framework: epidemiologists' quantitative, population‐level accounts arguably follow Graunt's 17th‐century lead much as historians follow that of Thucydides. As such counts became more common across the globe, particularly with the spread of European empires, official proclamations reflected the methods and approach developed by Graunt and his successors. Declarations of the end of England's 19th‐century cholera epidemics were likewise made on observations of constant decreases in cholera cases, using comparisons with previous years, and in comparison to other forms of mortality.^38^

As a result, although a few select epidemics—such as SARS—have been declared ended when cases among humans ceased, most epidemics have not concluded with the end of the disease.^39^ Indeed, smallpox is the only human disease ever to have been eradicated. As those who were part of that effort candidly stated, its eradication was a “rare event.”^40^ Guinea worm (*Dracunculus medinensis*), malaria, polio, yaws, and hookworm, among others, have been the target of longstanding eradication campaigns. All of these campaigns have either been downgraded to local campaigns to “control” the disease, or have had their target dates extended.^41^ Elimination of a disease at regional levels works for diseases of livestock because the movement of animals can be restricted (and culling can be implemented). But as virologists and public health specialists Walter Dowdle and Donald Hopkins point out, the inability to control human movement makes regional eradication an “oxymoron.”^42^ Moreover, the diseases targeted for eradication—smallpox, guinea worm, and polio—were and are not, strictly speaking, epidemic diseases. Instead, they were generally at endemic levels in the countries identified for the eradication. Indeed, many eradication campaigns have faced overwhelming obstacles because they identify and frame their target diseases as urgent problems, whereas the societies and governments in question accept them as endemic, and thus “normal” (if unpleasant) parts of life, compared to more pressing crises.^43^

Given such complexities, the resumption of normal patterns of life can be a more useful marker for the end of an epidemic than biomedical metrics. In medieval Europe, for example, the return of Jews and other groups that were often banished during outbreaks of disease signalled to contemporaries (and to historians) the end of a disease cycle. As the account of the Venetian plague by physician and rabbi Abraham Catalano (d. 1642) demonstrates, the end of an epidemic involved intense political and social negotiations because deep divides remained in communities that had viciously turned on those in their midst during the plague.^44^ For others, such as in early modern Italian city‐states or 18th‐ and 19th‐century empires, the resumption of markets and international trade signalled the end of an epidemic.^45^ Likewise, when the Danish king, at the end of the Baltic plague epidemic of 1710–1714, ordered that all bedding and beds of those who had been sick with the plague should be burned, many resisted; why, they asked, should they destroy valuable goods that they had used even as sickness and death rates fell? Their bewilderment and resistance indicate that for them, the epidemic had already ended and normal life had resumed; the royal decree was therefore an unreasonable (and expensive) intrusion.^46^ An official declaration, whether by a king or by an international body such as the WHO, is thus only one part of the ending of an epidemic and does not suffice on its own.

Epidemics, and particularly their endings, can thus only be understood within a broader context of continuing disease. Influenza A, with various strains in circulation throughout the world, is a classic example of a disease that has risen to epidemic—that is, problematic—levels before returning to endemic—or acceptable—levels in unpredictable waves ever since it first appeared among human populations in the late 1500s.^47^ Given that an epidemic is defined as an increase in incidence beyond usual rates, the end, too, can be clinically defined as the “reduction of disease incidence, prevalence, morbidity or mortality to a locally acceptable level” to achieve what is widely described as “disease control.”^48^ Yet as ambiguous terminology such as “acceptable” and “control” demonstrates, this status is necessarily achieved through a process of negotiation between different, if not competing, interests—particularly as historical examples demonstrate that an epidemic's end can be gauged by the resumption of social and economic practices as much as through biometrics. Indeed, the very process of an end highlights competing approaches. In discussions on the ending of HIV/AIDS, for example, debates as to whether it is more effective to invest in vaccine research, medical therapeutics such as antiretroviral therapy, or social and political reforms articulate different methodological understandings of the epidemic.^49^ Such divergent approaches also reflect different global circumstances: what might be feasible in communities with sophisticated health structures cannot be replicated in those confronting limited and unstable medical support, if not political fractures as well. What is deemed a “locally acceptable level” is thus necessarily a process of social and political debate as much as it is about disease.

## 3 PERSPECTIVES AND ENDS

Many of those subject to the risks and ravages of epidemic have little say in these debates, whether those who had no bedding to burn after the 1714 plague or those not privy to political negotiations over funding and declarations. Many are not even aware of such decisions in the midst of continuing disease and the upheavals it can bring. Those living through an epidemic may not even conceptualize it as a coherent outbreak; the first cholera pandemic, which arose in India in 1817, was described there at the time only as a “disorder,” as Mark Harrison explains. The term “epidemic” was applied to the outbreak only years later, in the context of an argument about the location (and not necessarily the virulence) of disease, “imposing a structure and unity that had not been obvious to most of those who had witnessed it.”^50^

While distance can help reveal coherence and patterns, shifting perspective can also render the definition of an end more complex. In the case of the Second Plague Pandemic, for example, a shift in temporal and geographic distance demonstrates that the end of an epidemic can be redefined according to a narrator's location, methodology, and periodization. Starting roughly in the 14th century and ending in the 19th century, the Second Plague Pandemic consisted of constant waves and cycles of *Yersinia pestis* that continued to circulate throughout Africa, Europe, and Asia through well‐entrenched trade routes and networks of communication. Spread via rodent populations such as rats, as well as insect vectors such as fleas, the plague has long been considered the standard model for understanding epidemic disease. It also serves as an historical model for the relationship between disease and society in general. And, although it was only recently granted coherence as a single pandemic by historians and biologists, it is one of the few epidemics whose ending scholars have discussed extensively.^51^

Plague was not a constant between ca. 1350 and ca. 1830. Rather, it was an expected (if unwelcome) regular visitor. Across those 400 years, historian Nükhet Varlık remarks, “living with the plague became a fact of life for many societies of Afro‐Eurasia.”^52^ After an initial epidemic with extremely high mortality rates—some estimates reach 50% of the entire population—local and regional epidemic outbreaks of plague regularly circulated throughout Afro‐Eurasia, with a total of perhaps 25 epidemics in all. While historians agree that these epidemics devastated populations and economies, their long‐term effects (including state centralization, technological efficiencies, and long‐distance migration) are widely debated.^53^

In Europe, the plague finally did recede: beginning in the late 1600s, its repeated waves slowed down until the last major wave—in 1720, in Marseille—marked the last plague outbreak in Western Europe. Scholars still debate why plague disappeared. Improved nutrition and thus increased immunity is one suggestion; improvements in sanitation and building works that would have affected vectors such as rats and fleas is another; still others suggest biological shifts in rat populations or related vectors.^54^ This debate has expanded to factor in climatic changes, the nature and movement of the plague through the field of phylogenetics, the role of other rodents, and the likelihood that fleas alone—not rats—were responsible for transmission.^55^ Historians have long insisted that beyond these environmental factors, human agency—administrative methods of quarantine, *cordons sanitaires*, port surveillance—was crucial to the decline of plague.^56^ But these options are by no means mutually exclusive; no single element was likely both necessary and sufficient, and indeed, they seem to have interacted with each other in ways as complex as the factors in the spread of disease. What recent research points to is the continued mystery behind the plague's decline: even with further data from a range of disciplines, it is unclear why the disease decreased in virulence or frequency. As a recent genomic synthesis paper points out, “the true processes are likely part of a complex web of dynamics, of which the available data only allow us to elucidate the most general trends.”^57^

Yet as Varlık has pointed out, focusing on plague's end in Western Europe obscures the fact that plague did not disappear from the rest of the world. Indeed, it is more accurate to suggest that plague simply went into abeyance in a few select countries for a short period of time, while remaining active within rodent populations, where it remains endemic today, and with scattered human cases regularly reported.^58^ More significant is that this undue focus on plague's remission in Europe encouraged Western epidemiologists—part of a discipline forming itself during this intermission of plague—to develop an optimistic belief in human ability to control, if not conquer, disease.^59^ If narratives of an epidemic's end focused not on human agency but on the role of ecology, climate, and even chance, and if they were written from a non‐European perspective—perhaps even a non‐human perspective—what might emerge is a continuous cycle of disease: a world that has continuously lived with plague, adapting to its movements, rather than a linear narrative of outbreak, increase, decline, and end of epidemics.^60^

Detailed scientific and historical scholarship on cholera suggests that similar revisions to its standard linear narrative are also in order. Popular accounts tend to focus on India as the origin of the “Asiatic cholera,” with London's Broad Street pump and the towering figure of John Snow (another “father of epidemiology”) as its dramatic ending. In this narrative, cholera was ended by the rise of laboratory medicine and bacteriology, symbolized by Snow's thorough statistical exercise in mapping cholera cases and, thereby, their source in south London water supplies.^61^ Like the traditional story of plague, this account of cholera suffers from significant geographical bias. Particularly from the standpoint of the Global South, cholera has not ended. It is a continuing disease associated with wars, political fractures, and economic upheavals.^62^ Recent scientific research has demonstrated that it is naturally, even fundamentally, part of lacustrine and coastal marine ecosystems; the bacteria “are marine organisms with permanent niches all over the world,” with accompanying endemicity.^63^

As a result, an end can be identified from the immediate vicinity of an individual epidemic. But from a perspective that ranges globally as well as across a longer timeframe, each ending begins to blur into a multiplicity of ends, if not repeated waves. For example, the 1918–1919 influenza pandemic has been divided into three “waves,” with some suggesting that the third wave “was really just a normal series of ‘trailer’ outbreaks.”^64^ What if, as anthropologist Christos Lynteris asks, every end is merely a hiatus, “‘before the return’ of an epidemic”?^65^ Would such a cyclical narrative be a more accurate model for the behaviour of epidemics? The Second Plague Pandemic was itself composed of a number of individual epidemics and outbreaks, each with its own regional end. Only from the vantage point of the 20th century did it come to be considered the precursor to the Third Plague Pandemic (ca. 1850–ca. 1960). As Andrew Price‐Smith argues, when it comes to disease, “Perhaps we have, as a species, overestimated our capacities to master the natural world and bend it to our will.”^66^

Narratives of increase and decline, outbreak and end, may not only falsely suggest humans' ability to conquer disease, they also outline a linear account of the disease itself, as identified by Rosenberg. Such narratives also isolate disease from its ecological setting. Long before COVID‐19's entanglements with comorbidities, researchers have pointed out that disease fundamentally interacts with other diseases, and epidemics too are fundamentally shaped by concurrent and previous patterns of disease, along with societal frameworks and practices connected to memories and fears of disease.^67^ As P. Wenzel Geissler and Ruth J. Prince observe, COVID‐19 in Kenya is best described not through a linear or cyclical narrative, but as part of “one long epidemic,” arriving alongside recent and ongoing outbreaks of Ebola, cholera, HIV/AIDS, tuberculosis, and cancer.^68^ Studies over a longer timeframe—such as the mapping of influenza epidemics across the entire 20th century that has revealed the interaction of various strains—may provide even more insights along these lines.^69^

## 4 CONCLUSION

Epidemics end once the diseases become accepted into people's daily lives and routines, becoming endemic—domesticated—and accepted. Endemic diseases typically lack an overarching narrative because they do not seem to require explanation. More often, they appear as integrated parts of the natural order of things. By contrast, epidemics—like the recurring narratives they produce—throw a society's confusion, fears, and anxieties into high relief. But as a result, epidemics and their narratives can also act to conceal the thickets of disease in which we live, including those lowly and constant problems of heart conditions, acute diarrhoea, and respiratory infections.

Focusing on how epidemics end is not simply a conceptual exercise, a narrative trick in which one views disease from a new perspective. Instead, it reveals the social and political processes by which a disease becomes endemic—that is, accepted—as well as who participates in that process and who is excluded. Moreover, a society's understanding of an epidemic is different at the beginning and climax of an epidemic than it is at the end of an epidemic. When communities are thrown into panic and turmoil by the outbreak of a new disease, when medical committees are convened and central governments spring into action, epidemics are understood in clear biological terms. Likewise, the hunt for a carrier, the identification of a pathogen, and the investigation of its mode of transmission focus on the biological nature of the disease.^70^ But at the end stages of epidemics, the disease is regarded through the filter of political, social, and economic dislocation—dislocations that have deepened as the epidemic progressed—articulating the processes by which policy decisions are debated and implemented, and the accommodations between scientific models and human behaviour. When epidemics end, emergency response teams leave and governments direct their attention to more pressing problems.^71^ Local communities revive normal patterns of life, dealing not only with the epidemic's repercussions, but also its aftershocks. Thus, the epidemic can be viewed as having ended even as dislocation, cases, and fatalities persist.

Linear narratives of epidemics surmise a single ending, rather than capturing cycles, waves, and the multiplicity of endings. The multidisciplinary approach suggested encourages researchers and policymakers to apply the insights visible at the ends of epidemics to the outbreaks and climaxes of epidemics. Directing attention to the end of epidemics not only offers valuable insights about the nature of epidemics, but also allows societies to consider the multiple ways in which epidemics can end. This opens up a broad range of possibilities in defining an end while an epidemic is still in progress. For COVID‐19, this focus on the multiple ends of epidemics explains the range of global approaches to the disease. While some countries, such as Japan, implemented a model to “live with” the disease without eradicating it, others, such as New Zealand, have pursued an “elimination strategy.”^72^ The vast range of responses to COVID‐19 reveals the range of understandings in different cultures—based on innumerable environmental, societal, historical, logistical, and diplomatic factors—of when normal life can resume. Understanding the end of epidemics as a process in which people, communities, and societies return to normalcy incorporates evolving approaches to disease, particularly as fluctuating belief in the ability to control, if not eradicate, a disease is also part of an epidemic's unfolding. Coupled with the humbling recognition that researchers are still ignorant as to why diseases recede or disappear, this framework for examining the ends of epidemics directs one's attention to the broader context and longer trajectories of disease, allowing a re‐examination of epidemics, their history, as well as their future.

## Footnotes

^1^ Christakis (2020); Denworth (2020); Evers (2020); Greene & Vargha (2020b); Kolata (2020); Honigsbaum (2020); White (2020).

^2^ Malki et al. (2020); Luo (2020).

^3^ AstraZeneca (2020); University of Oxford (2020).

^4^ Brannigan (1981); Latour (1987). See also Kermode (1967) on broader notions of “ending.”

^5^ This is part of a multidisciplinary network project, “How Epidemics End” (https://epidemics.web.ox.ac.uk/).

^6^ Rosenberg (1989); Slack (1996); Chigudu (2020); Engelmann (2020); Charters & McKay (2020).

^7^ Rosenberg (1989, p. 2); see also Rosenberg (1992).

^8^ Evans (1987).

^9^ Greene & Vargha (2020a, p. 36); see also Vargha (2016).

^10^ Page (1953); Longrigg (1980); Wylie & Stubbs (1983); see, for example, Cohn (2018).

^11^ Slack (1996, p. 9). Emphasis added.

^12^ See the sole mention to plague in Thucydides 5.16, after which it is not discussed.

^13^ Rosenberg (1962); Crosby (1976).

^14^ Frandsen (2010).

^15^ For example, Loh (2004); Abramowitz, Hipgrave, Witchard, & Heymann (2018).

^16^ Parag, Donnelly, Jha, & Thompson (2020, p. 16).

^17^ Parag et al. (2020, p. 3).

^18^ Chowell, Sattenspiel, Bansal, & Viboud (2016); Wearing, Rohani, Keeling, & Ellner (2005); Mollison (1995); Christakos, Olea, Serre, Yu, & Wang (2005).

^19^ U.S. Department of Health and Human Services (2006, p. I‐76); World Health Assembly (2008, p. 14, art. 12).

^20^ Mack, Snair, & Mundaca‐Shah (2016, p. 34).

^21^ Vetter, Kaiser, Schibler, Ciglenecki, & Bausch (2016).

^22^ Greene & Vargha (2020a); Vargha (2016); Nishiura, Miyamatsu, & Mizumoto (2016); Peckham (2016b).

^23^ Lancet Infectious Diseases (2016, p. 507). On not declaring PHEIC soon enough, see Cohen (2019). See also Eccleston‐Turner & Kamradt‐Scott (2019); Gostin et al. (2016); Farmer (2020); Prince (2019); Nguyen (2019).

^24^ Mack et al. (2016); Farmer (2020); Lancet Infectious Diseases (2016); Vetter et al. (2016); Korkoyah & Wreh (2015).

^25^ World Health Assembly (2008, art. 12); Durrheim, Gostin, & Moodley (2020).

^26^ World Health Organization (2019).

^27^ World Health Organization (2015a; 2015b; 2015c).

^28^ World Health Organization (2016a).

^29^ Giahyue (2016). See also Shrivastava, Shrivastava, & Ramasamy (2017).

^30^ World Health Organization (2016b). See also Thompson, Morgan, & Jalava (2019); Djaafara et al. (2020).

^31^ World Health Organization (2010a, 2010b).

^32^ Shoshan & Panzac (2006, p. 2); White (2010, p. 556); but see Conrad (1982).

33 Frandsen (2010, ch 7).

34 “Privy Council” (1849); “National Thanksgiving” (1849).

35 “Health of London” (1849); “On Settling the Week's Account” (1849).

36 Pelling (2020).

37 Graunt (1986, pp. 365–369).

38 Santillana et al. (2018); Nishiura (2016); Eyler (1979); Pelling (2020).

39 Nishiura (2016); McLean (2005). Smith? (2019) observes that the elimination of SARS can only be correlated, and not directly attributed, to human intervention; see also Peckham (2016b).

40 Arita, Wickett, & Nakane (2004, p. 1); see also Bhattacharya (2006). The animal disease rinderpest was also eradicated in 2001.

41 Dowdle & Hopkins (1997). In 1997 Dowdle and Hopkins had assumed Guinea worm was about to be eradicated, but this has since been revised to 2030: Roberts (2019).

42 Dowdle & Hopkins (1997, p. 11). Bovine tuberculosis in the UK also highlights the problem in multi‐animal disease eradication: Cassidy (2019).

43 Dowdle & Hopkins (1997).

44 Crown (1973); Einbinder (2018); Cohn (2007).

45 Cipolla (1981); Harrison (2012).

46 Frandsen (2010, pp. 411, 415).

47 Patterson (1986); Méthot & Alizon (2015); World Health Organization (2010a, 2010b).

48 Dowdle & Hopkins (1997, p. 28); Dowdle (1998, p. 23); Heymann (2006); Pelling (2020); see also U.S. Department of Health and Human Services (2006, p. I‐76); World Health Assembly (2008, p. 14, art. 12).

49 Mukherjee et al. (2003); Fauci, Folkers, & Marston (2014); Padma (2010); Poku (2016); Condon & Sinha (2008); Pogge, Rimmer, & Rubenstein (2010); Galvani, Pandey, Fitzpatrick, Medlock, & Gray (2018).

50 Harrison (2020, p. 543).

51 For example, Little (2007); Echenberg (2003); Green (2014); Sallares (2006).

52 Varlık (2020a, p. 6).

53 Cohn (2008); Belich (2016); Byrne (2012); Valles et al. (2020); Green (2014); Cantor (2001).

54 Hirst (1953); Appleby (1980); Slack (1981); Antoine (2008). The McKeown thesis and the related concept of the epidemiological transition are also relevant: McKeown (1976); Colgrove (2002); Omran (1971).

55 Whittles & Didelot (2016); Dean et al. (2018); Spyrou et al. (2016); Benedictow (2016).

56 For example, Slack (1981); Frandsen (2010).

57 Guellil et al. (2020).

58 Varlık (2019); Cohn (2008); Shakow (2010); Valles et al. (2020).

59 Varlık (2020a, 2020b).

60 On ecological and equilibrium approaches to disease, see Kunitz (1987); Kiple (1993); Mendelsohn (1998); Whitman (2000); W. Anderson (2004).

61 See Pelling (1978); McLeod (2000); Vinten‐Johansen et al. (2003) as correctives to these assumptions.

62 Chigudu (2020).

63 Echenberg (2011); Hamlin (2009); Morris (2011). See also McCann (2015) on malaria's ecology and historical persistence.

64 Patterson & Pyle (1991, p. 4).

65 Lynteris (2019, p. 88).

66 Price‐Smith (2002, p. 184).

67 Echenberg (2003); Peckham (2016a).

68 Geissler & Prince (2020, p. 253).

69 Méthot & Alizon (2015); Kilbourne (2003); MacPhail (2014).

70 For example, R. Anderson (2020); Chigudu (2020, esp. Chs. 3–4); Gostin, Lucey, & Phelan (2014). McKay (2017); Vandenbroucke & Pardoel (1989); Archambeau (2019).

71 Greene & Vargha (2020a); Farmer (2020).

72 Kazuto (2020); Du (2020); Baker, Kvalsvig, & Verrall (2020).
